# IMAGE QUALITY AND POTENTIAL DOSE REDUCTION USING ADVANCED MODELED ITERATIVE RECONSTRUCTION (ADMIRE) IN ABDOMINAL CT - A REVIEW

**DOI:** 10.1093/rpd/ncab020

**Published:** 2021-03-29

**Authors:** B Kataria, J Nilsson Althén, Ö Smedby, A Persson, H Sökjer, M Sandborg

**Affiliations:** Department of Radiology, Linköping University, Linköping, Sweden; Department of Health, Medicine & Caring Sciences, Linköping University, Linköping, Sweden; Center for Medical Image Science & Visualization (CMIV), Linköping University, Linköping, Sweden; Department of Health, Medicine & Caring Sciences, Linköping University, Linköping, Sweden; Department of Medical Physics, Linköping University, Linköping, Sweden; Department of Biomedical Engineering and Health Systems (MTH), KTH Royal Institute of Technology, Stockholm, Sweden; Department of Radiology, Linköping University, Linköping, Sweden; Department of Health, Medicine & Caring Sciences, Linköping University, Linköping, Sweden; Center for Medical Image Science & Visualization (CMIV), Linköping University, Linköping, Sweden; Department of Health, Medicine & Caring Sciences, Linköping University, Linköping, Sweden; Department of Health, Medicine & Caring Sciences, Linköping University, Linköping, Sweden; Center for Medical Image Science & Visualization (CMIV), Linköping University, Linköping, Sweden; Department of Medical Physics, Linköping University, Linköping, Sweden

## Abstract

Traditional filtered back projection (FBP) reconstruction methods have served the computed tomography (CT) community well for over 40 years. With the increased use of CT during the last decades, efforts to minimise patient exposure, while maintaining sufficient or improved image quality, have led to the development of model-based iterative reconstruction (MBIR) algorithms from several vendors. The usefulness of the advanced modeled iterative reconstruction (ADMIRE) (Siemens Healthineers) MBIR in abdominal CT is reviewed and its noise suppression and/or dose reduction possibilities explored. Quantitative and qualitative methods with phantom and human subjects were used. Assessment of the quality of phantom images will not always correlate positively with those of patient images, particularly at the higher strength of the ADMIRE algorithm. With few exceptions, ADMIRE Strength 3 typically allows for substantial noise reduction compared to FBP and hence to significant (≈30%) patient dose reductions. The size of the dose reductions depends on the diagnostic task.

## INTRODUCTION

Modern computed tomography (CT) scanners are equipped with several dose reduction features such as tube current modulation, automatic tube voltage selection, filtration, dynamic shielding and post-processing methods such as iterative reconstruction (IR)^(1)^. Due to lack of computational power, the implementation of IR in clinical applications was not possible in the infancy of CT. The faster real-time analytical reconstruction method filtered back projection (FBP), which has been the clinical standard for the past 40 years, has reached its limitation and does not allow for further dose reductions. The increasing use of CT in clinical practice and associated absorbed dose to the population have raised concerns about the adverse effects of ionising radiation. This has led to the introduction of several generations of vendor-specific IR algorithms between 2008 and 2015; their function and mechanism are based on the properties of the imaging system. The acronyms, key distinctive features as well as the year of introduction of these IR algorithms have previously been described by Qiu *et al*.^(2)^ and Aurumskjöld^(3)^. The function of IR is to improve image quality obtained primarily through reduction of noise while preserving spatial resolution and image contrast^(4)^. There are two main groups of IR algorithms: the statistical/hybrid IR and model-based IR (MBIR) algorithms. The statistical/hybrid algorithms mainly reduce noise while the MBIR algorithms, in addition to their denoising properties, also correct for image degrading effects by incorporating several geometric, optic and system models^(1,2,4,5)^. The strengths and weaknesses of noise reduction strategies are discussed by Ehman *et al*.^(5)^ in their comprehensive overview and review of qualitative and quantitative tools used in evaluation of noise reduction techniques in abdominopelvic CT.

Major CT vendors offer MBIR today^(3)^. The advanced modeled iterative reconstruction (ADMIRE) MBIR was introduced in 2014 by Siemens Healthineers. It is available in five strengths, where the proportion of noise reduction increases with increasing strength^(4)^. The dose reduction potential of ADMIRE can be mostly attributed to the decrease in image noise with increasing ADMIRE strength. However, there is some loss of information as non-linear effects of the algorithm alter the image structure when using higher strengths of the algorithm^(6)^. ADMIRE is a statistical IR method that, with its advanced regularization loop operating in a 3D voxel neighbourhood, separates noise from actual anatomical structures thus preserving the natural anatomical texture appearance^(7)^. ADMIRE has the reconstruction times almost equivalent to those of FBP, which facilitates its implementation in clinical practice^(7)^.

Since IR has become the clinical standard for image reconstruction in modern CT scanners, it is feasible to reduce radiation dose to the patients without compromising the image quality. The purpose of this paper is primarily to evaluate the performance of ADMIRE in abdominal CT by reviewing current published literature and to discuss the methodology used to assess image quality and potential dose reduction.

## MATERIALS AND METHOD

A database search was performed for papers published between 2014 and 2020, using the keywords iterative reconstruction, model-based iterative reconstruction, Advanced modeled iterative reconstruction, ADMIRE, image quality and potential dose reduction. Inclusion criteria were image quality and dose reduction assessment studies performed using ADMIRE in abdominal CT. All other articles were excluded. Thirteen studies are included in this review, seven of which evaluated the performance of ADMIRE in human subjects and six in phantom studies.

A brief description explaining function of the ADMIRE algorithm is provided below. A more detailed description of the basic principles of the algorithm is available in a white paper by Ramirez-Giraldo *et al*.^(7)^.

### Advanced modeled iterative reconstruction

**Figure 1:**
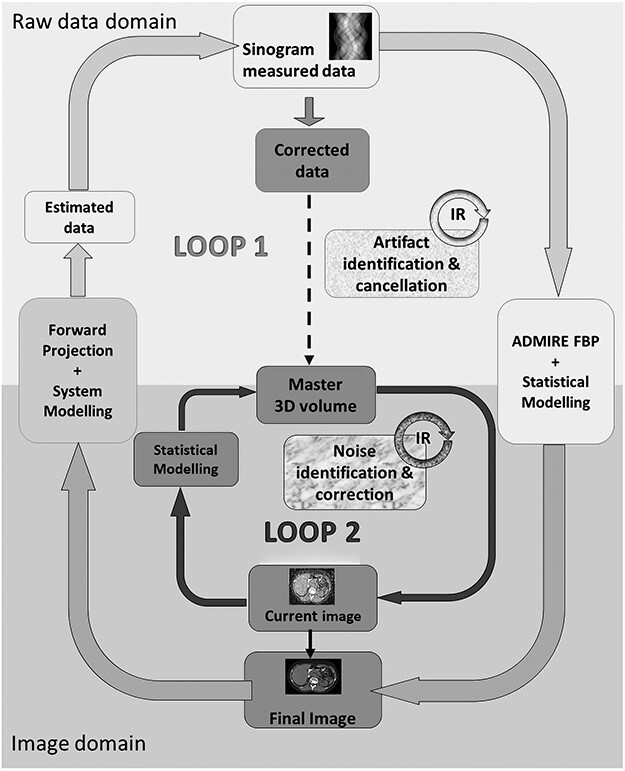
Basic principles of ADMIRE according to Ramirez-Giraldo *et al*., White Paper, ADMIRE advanced modeled iterative reconstruction^(7)^*.* Reproduced and altered/adapted with permission from Siemens Healthineers.

ADMIRE reconstruction implements two iterative loops during the reconstruction process; the first loop starts with a limited number of iterations in the raw data domain using statistical weighting primarily to reduce cone-beam artefacts and to a lesser extent noise. The second loop consists of iterations that reduce noise by means of statistical modelling performed in the image domain. The iteration process is speeded up as consequent iterations compare ‘current data sets’ with the master 3D volume, rendering the computationally intensive backward and forward projections unnecessary^(1)^ (Figure 1).

## RESULTS

The reviewed studies are divided into two study groups depending on the type of study (human and phantom) and are presented chronologically under each group according to the year of publication, starting with the oldest first. As acquisition parameters have bearing on the amount of potential dose reduction and comparison, a summary of the acquisition parameters for each of the studies included in the present evaluation of ADMIRE are presented in Table 1.

**Table 1:** Acquisition parameters for each of the studies included in the review.

Author study subject	Scanner type	kV	Qref mAs	Care dose	Care kV	Kernel	Collimation	Pitch	Rotation (s)	Slice thickness (mm)	Algorithm
Gordic *et al*.^(8)^ Human	Definition AS+	120 LD 100	289 60	Yes Yes	Yes No	B31f	128 × 0.6	0.9	—	5	FBP AD 1–5
Schaller *et al*.^(9)^ Human	Definition Edge	120(100)	210	Yes	Yes	B30		0.9	0.5	1, 3, 5	FBP AD 1, 3, 5
Ellman *et al*.^(6)^ Human	Definition AS+	120 (100)	289	Yes	Yes	B31f I31f	128 × 0.6	0.9	—	5	FBP AD 3, 5
Kataria *et al*.^(10)^ Human	Somatom Force	120	FD140 A 98, B 42	Yes	Yes	Br36	192 × 0.6	0.6	0.5	3	FBP AD 3, 5
Kataria *et al*.^(12)^ Human	Somatom Force	120	A 98, B 42	Yes	Yes	Br36	192 × 0.6	0.6	0.5	1, 2, 3	FBP AD 3, 5
Choi *et al*.^(13)^ Human	Somatom Force	100	FD150,LD100, ULD50	Yes	No	Br64	192 × 0.6	1.15	0.5	3	AD 2
Woisetschläger *et al*.^(14)^ Human	Somatom Force	70	(mA)150	—	—	Bv40	192 × 0.6	—	—	1.5	FBP AD 3, 4, 5
Solomon *et al*.^(15)^ Phantom	Somatom Force	120	11,21 43,87	Yes	No	Bf44	192 × 0.6	—	—	0.6, 1.8, 5	FBP AD 3–5
Ott *et al*.^(16)^ Phantom	Somatom Force	120	1,3,5,8,15 CTDI_vol_	—	—	—	192 × 0.6	0.98	—	2	AD 1, 3
Euler *et al*.^(17)^ Phantom	Somatom Force	70,80,100 (ref)120	4 dose indices kV/phantom size	Yes	Yes	Br40 I40	192 × 0.6	0.8	0.5	5	FBP AD 3
Dalehaug *et al*.^(18)^ Phantom	Somatom Flash	120	150 LD 30	No	No	B30f	192 × 0.6	—	—	—	FBP AD 1, 3, 5
Alikhani *et al*.^(19)^ Phantom	Somatom Force	120	(mA) 20,50 100, 200 (ref) 900	No	No	Bf44		1	0.5	1	FBP AD 1–5
Viry *et al*^(.20)^ Phantom	Definition Edge	120	130 *88, 125, 182	—	—	B30f I30f	128 × 0.6	0.8	0.5	2	FBP AD 3

AD = ADMIRE, FBP = filtered back projection, FD = full dose, LD = low dose, ULD = ultra-low dose, ref = reference, Qref = quality reference, A = tube A, B = tube B, *Effective mAs.

### Literature review

Tables 2 and 3 provide a summary of the studies included in the literature review outlining the main findings, evaluation methods and type of study performed.

**Table 2:** Summary of the evaluation methods and main findings in human studies included in the literature review assessing image quality and dose reduction potential of ADMIRE algorithm in abdominal CT. Diagnostic accuracy studies appear in bold text.

Authors	Evaluation methods		Main findings
	Subjective	Objective	
Gordic *et al*.^(8)^	Image noise, artefacts, visibility of small structures, image contrast.	Attenuation at six anatomical sites. Image noise.	Improved image quality with increasing AD strength (AD1→ AD5) allowing for substantial noise reduction (8–53%). Retained attenuation in all anatomical regions for all algorithms.
Schaller *et al*.^(9)^	Detectability and conspicuity of low-contrast lesions.	Attenuation at two anatomical sites. Differences and detail preservation in subtraction images.	Noise reduction of 8.5–54.4% for AD1, 3 and 5 compared to FBP depending on slice thickness. No obvious negative impact on lesion depiction. No relevant detail loss in IR process.
Ellman *et al*.^(6)^	Observer rating of anatomical structures for three high-contrast (HC), medium-contrast (MC), low-contrast (LC) groups.	Attenuation based contrast grouping of anatomical structures. Noise.	Noise reduction increased with AD strength. Dose reduction ranged from 29 to 53.5% depending on object contrast with no significant difference between AD3 and AD5. AD5 has no advantage over AD3.
Kataria *et al*.^(10)^	Observer rating of six image criteria.	–	Dose reduction of 30% without change in algorithm, additional reduction AD3 22–47% (all criteria), AD5 34–74% (some criteria).
Kataria *et al*.^(12)^	Observer rating of five image criteria.	SNR, CNR and NPS.	Dose reduction 24–41% with increasing slice thickness from 1 to 2 or 3 mm. AD3 showed improved image quality for all criteria. AD5 only two criteria out of five.
**Choi *et al***.^(13)^	**Observer evaluation of lesion characterization and conspicuity, diagnostic confidence in five organs.**	**Image noise and SNR.**	**At 30% dose reduction, AD2 produced images of acceptable image quality at high specificity, sensitivity. At 60% dose reduction, image quality was suboptimal.**
Woisetschläger *et al*.^(14)^	–	Quantitative time attenuating curve measurements, Image noise and SNR.	Lower noise and higher SNR with increasing AD strength (AD1 → AD5) helped to preserve image quality in CTP.

AD = ADMIRE, FBP = filtered back projection, IR = iterative reconstruction, SNR = signal-to-noise ratio, CNR = contrast-to-noise ratio, NPS = noise power spectrum. **Bold text = diagnostic accuracy study**, CTP = CT perfusion.

**Table 3:** Summary of the evaluation methods and main findings in phantom studies included in the literature review assessing image quality and dose reduction potential of ADMIRE algorithm in abdominal CT. Diagnostic accuracy studies appear in bold text.

Authors	Evaluation methods		Main findings
	Subjective	Objective	
**Solomon *et al***.^(15)^	**Detection accuracy of virtual objects.**	**Resolution, contrast, number of visible objects, NPS, TTF AUC and detection accuracy.**	**5.2% higher detection accuracy for AD compared to FBP. Dose reduction 56–60% for AD3 compared to FBP. 4–80% (mean 41%) dose reduction for AD3–5 compared to FBP depending on slice thickness, reference FBP and AD strength.**
**Ott *et al***.^(16)^	**4-AFC human observer low-contrast detail detection.**	**LCD by CHO-model observer.**	**Improvement in low-contrast detection with AD. Increase in PC scores with increasing AD strength.**
Euler *et al*.^(17)^	Grade of lesion conspicuity, image noise, image quality assessment with kV variation.	Lesion to background CNR.	AD decreased noise and increased CNR at different tube voltages. CNR increased by 16–58% in medium phantom and 9–18% in large phantom compared to FBP. No significant improvement in lesion detection between AD and FBP.
Dalehaug *et al*.^(18)^	–	Noise and NPS. Inter-image SD maps.	At low dose, AD1, 3 and 5 removed more noise compared to SAFIRE. At low dose, NPS curve shifted towards lower spatial frequencies. AD removed noise efficiently around edges.
Alikhani *et al*.^(19)^	–	Image texture (Haralick) SSIM, Noise, MTF	Maintained image texture for AD3, AD4 and AD5 at 50% dose reduction compared to FBP. SSIM for AD4 and AD5 was similar to FBP at 50% dose level. Retained spatial resolution with up to 90% dose reduction.
**Viry *et al***.^(20)^	–	**LCD by CHO-model observer**	**Limited improvement in LCD using AD3 compared to FBP.**

AD = ADMIRE, FBP = filtered back projection, SNR = signal-to-noise ratio, CNR = contrast-to-noise ratio, NPS = noise power spectrum. **Bold text = diagnostic accuracy study**, TTF = task transfer function, AFC = alternative forced choice, LCD = low-contrast detectability, CHO = Channelized Hotelling observer, PC = percentage correct, SD = standard deviation, SAFIRE = Sinogram affirmed iterative reconstruction, SSIM = structural similarity index, MTF = modulation transfer function.

#### Human studies

One of the first to evaluate the performance of ADMIRE was Gordic *et al*.^(8)^. They compared images reconstructed with FBP and all ADMIRE strengths. The study population consisted of 10 patients each undergoing a standard dose, at four different tube voltages, and a low-dose abdominal CT. Image quality was determined by qualitative assessment of image noise, artefacts, visibility of small structures, image contrast and quantitative measurements of objective image noise and attenuation at several anatomical sites by two independent readers. Image noise decreased and image contrast increased with increasing strength of the algorithm. Noise reduction of approximately 10% per ADMIRE strength level was found to be significant when compared to FBP. The conclusion was that ADMIRE improved subjective and objective image quality when compared to FBP.

Schaller *et al*.^(9)^ compared image quality between FBP and three ADMIRE strengths at three slice thicknesses to assess the potential for noise reduction in contrast-enhanced CT abdomen examinations. Objective noise was measured by placing multiple regions of interest (ROIs) in the liver and spleen and subjective image quality assessment was performed using a 5-point Likert scale. To visualise differences between FBP and ADMIRE and ascertain detail loss, subtractions of images at all strengths of ADMIRE from FBP images were performed. Potential image noise reduction of up to 50% was possible with no loss of relevant details in the iterative reconstruction process.

Ellman *et al*.^(6)^ used a propriety workstation (ReconCT) to reconstruct full dose images with FBP, ADMIRE Strengths 3 and 5 as well as simulated reduced dose ADMIRE data sets at 10% intervals to ascertain the degree of potential dose reduction. Pairwise comparisons of full dose FBP and reduced dose ADMIRE were performed using six anatomical criteria grouped into three intrinsic contrast subgroups (high, medium and low), and the dependence on radiation dose was analysed by studying observer preferences at different doses with non-linear regression. They also compared the noise reduction for the IR algorithm with the radiation dose reduction. Their results show that significant dose reductions are possible with no differences between ADMIRE 3 and 5 within contrast subgroups. However, for ADMIRE 3, there were significant differences in dose reduction between all of the three contrast subgroups, whereas for ADMIRE 5, this was true only between high- and medium-contrast subgroups. Potential dose reduction (DRP) was calculated by identifying the point at which there was no preference between full-dose and dose-reduced images (indecision point, IP) and then applying the formula $DRP=100\%- IP$. They concluded that a 30% dose reduction was achieved while maintaining image quality, lesion detectability and visual impression in abdominal CT using ADMIRE. Although ADMIRE 5 permits higher noise reduction, it does not enable corresponding higher levels of dose reduction. Therefore, ADMIRE Strength 5 has no concrete advantage over ADMIRE Strength 3.

**Figure 2:**
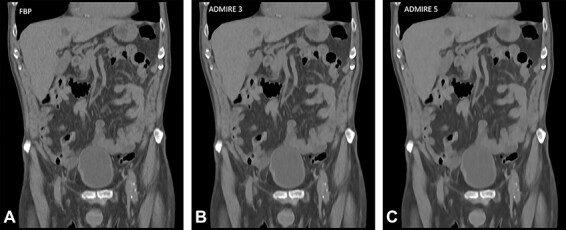
Examples of image quality comparisons in full-dose abdominal CT (quality reference (Qref) 140 mAs) from a study patient^(10)^, reconstructed with FBP and ADMIRE (Siemens Healthineers) at Strengths 3 and 5, out of 5 available strengths.

A pairwise comparison study performed by Kataria *et al*.^(10)^ compared FBP, ADMIRE Strength 3 and 5 to ascertain potential dose reduction using a dual-source CT scanner in the experimental mode to generate three data sets per patient at dose levels 30, 70 and 100%. Examples of images from a study patient showing the image quality at 100% dose level and reconstructed with FBP, ADMIRE Strengths 3 and 5 are presented in Figure 2. Independent readers performed visual grading assessment using six image criteria, and potential dose reduction was estimated using visual grading regression (VGR)^(11)^. VGR is an ordinal logistic regression model applied to scores from visual ratings, controlling for dependencies between observers, patients, tube loads and reconstruction methods. The results indicated that as there was no difference in image quality for doses of 70 and 100% of the standard setting; thus, a 30% dose reduction was possible without any change in algorithm. When comparing dose levels 30 and 70%, ADMIRE 3 produced images of superior quality in relation to FBP thereby facilitating a further dose reduction of 22–47% for all criteria assessed. ADMIRE 5, on the other hand, allowed for a further dose reduction of 34–74% for all criteria with the exception of Criterion 1, the liver parenchyma. They concluded that in relation to FBP, there is a positive correlation between potential dose reduction and ADMIRE strength for all but one image criterion.

Kataria *et al*.^(12)^ performed another visual grading experiment to study the effect of tube load, ADMIRE Strengths 3 and 5 and slice thickness on potential dose reduction in a pairwise comparison of multi-planar reconstruction images at two dose levels of 30 and 70%. Interpretation of both objective measurements of image noise, contrast-to-noise (CNR) ratio and noise power spectra (NPS) as well as subjective image quality assessments (determined by independent readers using image quality criteria) were used to explain the resulting improvements/degradation in image quality and feasible dose reductions. Increase in slice thickness and tube load correlated to improvement in image quality with possible dose reductions, regardless of the algorithm strength. ADMIRE Strength 3 consistently produced images of better quality for all criteria assessed when compared to ADMIRE 5, which had diverse effects on image quality. They concluded that ADMIRE 5 could not be recommended to replace ADMIRE 3 in clinical practice but can possibly be used in specific task-based protocols.

Choi *et al*.^(13)^ conducted a focal lesion detection study in contrast-enhanced abdominopelvic CT, comparing three data sets at tube loads 100% (full dose), 66.7% (low dose) and 33.3% (ultra-low dose). Their results showed a high sensitivity and specificity for all focal lesions in representative abdominal organs with acceptable image quality on low-dose CT with an effective dose of 2.6 mSv. The ultra-low dose with an effective dose of 1.3 mSv, however, produced images of suboptimal quality and lower sensitivity and specificity for focal lesions in almost all organs except for enlarged lymph nodes, which showed 100% sensitivity and accuracy. They concluded that ADMIRE Strength 2 allows for a 30% dose reduction in abdominal CT as the low-dose CT performs similar to a standard-dose CT. An ultra-low-dose protocol may be useful in evaluation of enlarged lymph nodes.

Woisetschläger *et al*.^(14)^ evaluated CT perfusion examinations of the upper abdomen to assess differences in image quality between FBP and ADMIRE Strengths 3, 4 and 5. Quantitative measurements of blood flow, blood volume and time to peak, arterial liver perfusion, portal venous liver perfusion and hepatic perfusion index were generated by placing identical sized ROIs in identical positions in the following tissues; left liver lobe, right liver lobe, hepatocellular carcinoma, spleen, gastric wall, pancreas and portal vein for all four reconstruction types using the maximum-slope model. These Hounsfield units (HU) measurements were performed in images reconstructed with temporal maximum intensity (TMIP) and temporal average (TAVG) projections. Image quality was assessed by comparing measures of noise (standard deviation (SD) of the ROIs) and signal-to-noise ratio (SNR) in each organ. The image noise was lower and the SNR was higher with increase in ADMIRE strength. Their results indicated that ADMIRE had no effect on the quantitative measurements or time-attenuation curves of the tissues assessed as no significant differences were found despite significant differences in image noise and SNR between the four reconstruction algorithms.

#### Phantom studies

Solomon *et al*.^(15)^ performed a contrast-detail phantom study to assess the effect of dose reduction on low-contrast detectability (LCD). A 3D printer-fabricated phantom was scanned in a dual-source scanner at four different radiation dose index levels, reconstructed with three different slice thicknesses and reconstruction algorithms FBP and ADMIRE Strengths 3, 4 and 5. LCD increased with increase in object size, contrast, slice thickness and ADMIRE strength. Potential dose reduction was calculated by fitting the observer data to empirical mathematical models. In the first reading session, a comparison between FBP and ADMIRE 3 allowed for 56–60% dose reduction depending on the reference FBP dose index. The second reading session compared FBP to ADMIRE Strengths 3, 4 and 5 with a dose reduction ranging from 4 to 80% depending on the reference FBP dose index, slice thickness and ADMIRE strength, while preserving LCD.

Ott *et al*.^(16)^ evaluated the performance of ADMIRE Strength 3 in an anthropomorphic phantom (QRM, Moehrendorf, Germany) with two custom made embedded centre modules; a homogenous module and a low-contrast module with spherical targets of 6 and 8 mm diameter at 10 and 20 HU contrast level compared to the surrounding material. A 4-alternative forced choice experiment was performed using three human observers, and a channelized hotelling observer (CHO) to detect signal images and the percentage correct (PC) responses were obtained. They concluded that the CHO model observer successfully reproduced the human observer’s response in low-contrast detection and that using ADMIRE (at Strength 3) led to improvements in PC compared to lower ADMIRE strengths and FBP particularly at the low CTDI_vol_ range. Their results suggest that patient doses could be reduced with ADMIRE but do not provide quantitative numbers for potential dose reductions.

In a phantom study simulating medium and large size patients with hypoattenuating lesions, Euler *et al*.^(17)^ evaluated image quality and low-contrast lesion detectability in images reconstructed with FBP and ADMIRE Strength 3 at four tube voltage levels (70, 80, 100 and 120 kV) and four effective mAs values for each phantom size. Forty-five hypodense lesions with diameters of 5, 10 and 15 mm and three different lesion-to-background contrasts (10, 20 and 50 HU) were assessed by two different radiologist groups for lesion conspicuity in the medium and large phantom data sets. Noise increased in the large phantom at 70 and 80 kV with both algorithms. When comparing ADMIRE to FBP, CNR increased with reduction in tube voltage ranging from 27.3 to 32.4% and 23.5 to 33.3% in the medium and large phantoms, respectively. Despite the improvement in objective quality parameters when comparing ADMIRE to FBP, no significant difference in overall low-contrast detection rate was observed, regardless of tube voltage setting or reconstruction algorithm.

Since statistical modelling in the projection and image domains have been improved in the model-based ADMIRE algorithm when compared to Sinogram-affirmed iterative reconstruction (SAFIRE), Dalehaug *et al*.^(18)^ quantitatively compared the noise reduction properties of these two different iterative reconstruction algorithms from the same vendor. The homogenous module of the Catphan phantom (The Phantom Laboratory, Salem, USA) was used to measure noise and to calculate NPS. Further, an anthropomorphic phantom was scanned at two different dose levels to calculate 2-D inter-image SD maps. The full-dose images produced similar median values of the NPS curves for both algorithms at all strengths. However, for the low-dose images, the median values shifted towards the lower spatial frequencies of the NPS curve and were more prominent for ADMIRE at all strengths compared to SAFIRE. The shift towards lower spatial frequencies usually results in a more ‘blotchy’ image texture. A more efficient noise removal around the edges was observed for ADMIRE in comparison to SAFIRE, based on the inter-image SD maps (comparisons of SD of each pixel in the scanned images for combinations of both IR types and strengths).

A unique phantom study performed by Alikhani *et al*.^(19)^ studied the impact of ADMIRE on image texture using the Haralick texture parameters (an analysis method based on correlations between grey-tone combinations of pixels) and visual impression using the structural similarity index (SSIM). SSIM is based on the analysis of the luminance, contrast and structural similarity of two images and provides a good approximation of perceived image quality. They also measured noise (by subtracting the images of the uniformity module of the ACR phantom from the gold-standard image (900 mA FBP)) and high-contrast resolution determined by the modulation transfer function. Results from their study show that 50% dose-reduced images with ADMIRE Strength 3 up to 5 produced comparable results to those with FBP regarding contrast and entropy. Similarly, comparison between all ADMIRE levels and FBP provided improved SSIM values for the MBIR. SSIM calculated values for 50% dose-reduced images reconstructed with ADMIRE 4 and 5 were comparable to full-dose FBP images. Retained spatial resolution was maintained for ADMIRE with up to 90% dose reduction. Considering noise distribution in the background images, the HU numbers shifted towards a narrower distribution at increasing dose levels. A similar HU distribution shift was observed for all ADMIRE levels.

Viry *et al*.^(20)^ used a task*-*based approach to quantitatively assess image quality in abdominal CT. They performed receiver operating characteristic (ROC) studies using three sizes of anthropomorphic abdominal phantoms (25, 30 and 35 cm in diameter) with low-contrast spheres and hypodense module inserts. The low-contrast module contained 24 spheres mimicking abdominal lesions at 8, 6, 5, 4 and 3 mm to assess image quality in both FBP and ADMIRE 3 reconstructed images. LCD was assessed by means of the area under the ROC curve (AUC) for three clinically relevant (8, 6 and 5 mm diameter) lesions in images reconstructed with the two algorithms in three phantom sizes. For all sizes of phantom, no significant improvement in terms of AUC was found for comparison between ADMIRE 3 and FBP concerning the smallest and most difficult low-contrast detail task (5 mm). However, for the larger lesions (6 and 8 mm), ADMIRE 3 showed a small significant improvement in AUC in the large phantom. Their recommendation was to focus on the diagnostic requirements, clinical task and body size when optimising protocols.

## DISCUSSION

Since its introduction in 2014, a variety of studies^(6,8–10,12–18,20)^ have assessed the effect and performance of ADMIRE both quantitatively and qualitatively using phantoms and human subjects. However, very few studies^(13,15,16,20)^ have evaluated the diagnostic accuracy (which is measured using ROC analysis) of the ADMIRE algorithm.

The methodology used in the reviewed studies includes objective measurements, phantom studies and studies involving visual lesion detection and visual grading of clinical images. The comparisons performed varied from absolute to pairwise comparisons of IR images to previous standard FBP or comparison of two different vendor-specific IR algorithms. Despite the variation in methodology, the authors have come to quite similar conclusions. It is known that subjective evaluations are prone to observer bias^(1)^ but are clinically more relevant than phantom studies. Therefore, choice of comparison type is important. Simultaneous viewing of images pairwise tends to increase the ability to identify subtle differences in image quality between the images^(1)^, which may not be apparent when viewing the images separately. On the other hand, separate image assessment would be a better choice in lesion detection studies where the image depicting the lesion best might otherwise influence the observer if pairwise comparisons are performed.

Dose reduction studies preferably require two or more data sets at different dose levels in the same patient. As observed in the literature review, this was accomplished in several different ways: (1) splitting the dose between the two x-ray tubes in a dual-source scanner^(10,13,18)^, (2) simulating reduced dose by adding noise to full-dose image material^(6)^ and (3) use of phantoms instead of human subjects^(15,17,18)^. The above methods help to overcome ethical issues concerning repetitive imaging in the same patient.

Image quality can be assessed both quantitatively and qualitatively: some of the reviewed publications have included both quantitative and qualitative assessments^(6,8,9,12,13,15–17)^ and some only quantitative^(14,16,18,20)^ or qualitative^(10)^ assessments. It is apparent from the review that noise reduction measurements cannot be directly transferred into potential dose reduction as IR algorithms are subject to change in noise texture particularly at lower radiation doses, which ultimately affects image quality^(15)^.

The NPS is an objective measure of noise texture. Other objective measurements such as SNR and CNR predict equal performance for images with equal contrast and noise magnitude despite the difference in noise texture. As IR algorithms affect noise texture, measurements of SNR and CNR may not be sufficient to evaluate the effect of these algorithms^(15)^. When using IR algorithms to optimise radiation dose, it is important to bear in mind that change in image appearance may not affect either detectability or visibility of the lesions but may result in lower diagnostic confidence. Therefore, both quantitative and qualitative assessments linked to a specific diagnostic task evaluation using human observers are necessary^(1,20,21)^.

The reviewed studies showed that potential dose reduction can be calculated in several different ways. Solomon *et al*.^(15)^ accomplished this by fitting the observer data to empirical mathematical models; Ellman *et al*.^(6)^ used a mathematical formula by subtracting the indecision point value from 100 and Kataria *et al*.^(10,12)^ used VGR^(11)^. VGR is an ordinal logistic regression where the parametric model provides direct estimations of dose reduction and allows for simultaneous analysis of several parameters (fixed effects) that potentially influence image quality^(22)^. Examples of such fixed effects are choice of equipment, acquisition settings and post processing methods used. Since the observer and patient identities are not of primary interest for the researcher, VGR lets the researcher control for such variations between individuals by treating them as random effects^(11,22)^.

It is difficult to compare studies evaluating low-contrast objects, as contradictory results are sometimes present, which may depend on the acquisition parameters, such as lower tube voltage settings or contrast enhancement, that affect image quality. The same applies to phantom studies compared to human studies, as the task of the reader is a lot simpler in assessing lesions in a phantom compared to the clinical radiologist’s *in vivo* assessment^(1)^. The potential for dose reduction should first be considered after evaluation of clinical image quality criteria that are linked to specific clinical tasks^(20)^. Viry *et al*. advised against using results from simple LCD evaluation studies to optimise clinical protocols, as the tasks performed in these studies are far too simple compared to the clinical reality. Since there are considerable noise texture differences between the reconstruction algorithms, it is difficult to draw relevant conclusions regarding human observer assessment of LCD and therefore adding complexity to the diagnostic task, i.e. correct location and assessment of size and shape of lesion might render better performance assessments of IR algorithms^(20)^. The few ROC studies found were performed in phantoms^(15,16)^ with the exception of one study in human subjects^(13)^. The results of phantom and model observer studies have limited clinical validity and hence the diagnostic accuracy of ADMIRE is not fully known.

## CONCLUSION

The ADMIRE algorithm is a useful tool to reduce patient radiation dose in clinical abdominal CT. With few exceptions, ADMIRE Strength 3 typically allows for substantial noise reduction compared to FBP and hence to significant (≈30%) patient dose reductions depending on the diagnostic task. To estimate potential dose reduction using ordinal regression models is an option, as they allow for simultaneous analysis of several parameters and provide direct dose reduction estimates.

## FUNDING

This work was supported by ALF-(LiO-602731, LIO-697941), FoU-(LIO-724631, LIO-620341) and RFoU- grants from Region Östergötland and the Medical Faculty at Linköping University.

## CONFLICT OF INTEREST

The authors declare no conflicts of interest with regards to this work.
